# The transition from bee-to-fly dominated communities with increasing elevation and greater forest canopy cover

**DOI:** 10.1371/journal.pone.0217198

**Published:** 2019-06-12

**Authors:** Lindsie M. McCabe, Ella Colella, Paige Chesshire, Dave Smith, Neil S. Cobb

**Affiliations:** 1 Colorado Plateau Biodiversity Center and Department of Biological Sciences, Northern Arizona University, Flagstaff, Arizona, United States of America; 2 U.S. Fish and Wildlife Service, Southwest Forest Science Complex, Flagstaff, Arizona, United States of America; University of New England, AUSTRALIA

## Abstract

Insect pollinator communities are thought to transition from bee-dominated communities at low elevations to fly-dominated communities at high elevations. We predicted that increased tree canopy cover and a subsequent decrease in meadows and flowering plants would limit bees but not flies at higher elevations. We tested and supported this prediction by examining changes in both abundance and species richness for 128 bee species and 96 fly species at key points along an elevational gradient in Northern Arizona represented by distinct vegetation life zones. In addition to an increase in fly species and abundance relative to bees with increasing elevation, there were changes in community structure). To better understand factors that might influence this transition we examined how tree canopy cover changed along the elevational gradient and how this influenced the change in insect pollinator communities. While bee communities were progressively divergent between forest and meadow habitats with increasing elevation and tree canopy cover, there was no significant pattern with flies between meadow and forest habitats. However, fly abundance did increase with increasing elevation relative to bees. Along a comparable elevational gradient on an adjacent mountain with no tree canopy cover (i.e., a fire burned mountain), the bee-to-fly transition did not occur; bees persisted as the dominant pollinator into the highest life zone. This suggests that tree canopy cover can in part explain the transition from bee-to fly-dominated communities. In conclusion, this is the first study in North America to document a bee-fly transition for both abundance and species richness and show that tree canopy cover may play a role in determining pollinator community composition, by restricting bees to open meadow habitats.

## Introduction

Bees and flies are the two dominant insect pollinators in almost every ecosystem [1]; however, they are thought to be differentially distributed along elevational gradients. It has been proposed that bees dominate pollinator communities at low elevations and are replaced by flies at higher elevations [2]. This pattern has been consistently observed in studies spanning numerous regions across countries from New Zealand and Australia [3], Nepal [4], Chile [5], Switzerland [6, 7], New Hampshire, USA [8], and British Columbia, CA [9]. However, most of these studies have only anecdotally observed this transition, and the few that have examined this transition [2] have addressed the phenomenon only in general terms (e.g., noting that flies are higher in abundance or richness than ‘normal’ but not directly comparing them to other taxa). No study to date has examined environmental factors beyond elevation that could explain this transition.

Bees are the most important group of pollinators for angiosperms [10–12] and are highly specialized herbivores, with adults and larvae feeding exclusively on nectar and pollen resources [13]. Most bee species are found in open habitats [14] where both floral resources and ground-nesting sites are more abundant compared to closed-canopy forests. While flies are not as efficient pollinators as bees, certain families of flies are known to provide vital pollination services [15, 16]. Syrphidae, Muscidae and Bombyliidae are considered the most prevalent fly pollinators [17–19]; however, there has been increased evidence documenting tachinid and muscoid flies as dominant pollinators in high mountain environments [3]. Fly dominated pollinator communities are most prevalent in high elevation/alpine environments [20] but they have also been documented to dominate along precipitation gradients, where moisture is greater [21], and in upper latitude grassland prairies [22]. Unlike bees, flower-visiting flies do not forage for pollen or nectar resources for their offspring [19, 23, 24]. Instead, fly larvae of flower-visiting adults typically function as foraging herbivores, decomposers of rotting organic matter, parasites of other insects [25], or, in very rare instances, pollen eaters [26]. Flower-visiting adult flies, however, do typically feed on nectar and in some cases pollen. Thus, flies occupy different niche spaces during adult and larval life stages than bees.

Tree canopy cover may play an important role in pollinator communities as elevation increased along mountains up to tree line. Dense tree crowns typically reduce or eliminate the flowering plants that provide nectar and pollen resources to the pollinator communities [27]. This is especially true for conifer forests, which not only reduce light to the understory, but also create highly acidic litter that typically prevents many flowering plants from growing [28]. Forest canopy cover increases as elevation increases, until tree line [29], therefore reducing the amount of meadow space and floral resources in higher elevation habitats [30]. Lower flower abundance at high elevations reduces the amount of pollen and nectar resources that bees require [31]. Although forest canopy cover is known to limit floral resources for bees, eliminating this resource is problematic for tree nesting species, such as Megachilidae, that rely on preexisting biotic cavities, such as dead and down trees, for their nesting resources [32]. Understanding the effect that canopy cover has on pollinator community structure may help us determine which bee species are the most specialized along elevational gradients.

This study focused on the elevational gradients on the San Francisco Peaks and the nearby Kendrick Mountain, which represents one of the more northern sky islands found in the Southwest. The San Francisco Peaks was the inspiration for the development of the life-zone concept [33], and has been a model for understanding ecological processes using an elevational gradient [34]. The San Francisco Peaks span seven distinct life zones: desert (~1600m), desert grassland (~1700m), pinyon-juniper (~2000m), ponderosa forest (~2400m), mixed conifer (~2600m), spruce-fir (~3000m) and alpine (~3600). For this study we focused on the three highest life zones below tree line (ponderosa, mixed conifer, and spruce-fir). We identified two primary goals; the first was to explicitly test the hypothesis that pollinator communities are dominated by bees at lower elevations and flies at higher elevations. Our second goal was to examine how changes in forest canopy cover can explain the changes in relative abundance and species richness of bees and flies. We specifically predicted that 1) bees would be less abundant and diverse, compared to flies, due to a reduction in meadow habitats as elevation increases. 2) Increasing forest tree canopy cover would reduce floral resources for bees, but not flies, and thus bees would be more constrained by canopy cover than flies.

## Methods

We conducted two complementary studies to examine changes in pollinator communities along elevational gradients resulting from: 1) elevation and tree cover effects in meadow and forest habitats of an unburned mountain, and 2) the effects of elevation in meadow habitats of a burned mountain.

### Study sites

Research was conducted along an elevational gradient on the north side of the San Francisco Peaks, AZ (35.334, -111.659). A total of nine sites were sampled at three different elevations, representing three forest types; ponderosa (~2400 m), mixed conifer (~2600 m) and spruce-fir (~3000 m) (Table 1). We refer to these three levels of elevation by the life zones that characterize those elevations. Our study areas were restricted to this range of elevations because they included the presumed bee-to-fly transition zone based on previous surveys [35]. The average distance between sites on the San Francisco Peaks was 1.50 +/- 0.38 kilometers. At each site we established one trap array in open meadows and one array in forest habitats. The distance between meadow and forest arrays ranged from 100–200 meters depending on the size of the meadow. The given distances between sites allow for independent sampling of pollinator communities, due to the average foraging range of the bees (~ 1 km) sampled in our community [36, 37]. Sampling was conducted on National Forest Service lands, with appropriate permits. Our study did not involve endangered or protected species.

**Table 1:** Locations and elevation of each of the nine sites per mountain (The San Francisco Peaks and Kendrick).

Mountain	Life Zone	Mean Elevation (+/- SE)	Site ID	Elevation (m)
San Francisco Peaks	Ponderosa	2374 (+/- 28)	PP1	2420
			PP2	2380
			PP3	2323
	Mixed Conifer	2601 (+/- 46)	MC1	2514
			MC2	2675
			MC3	2614
	Spruce-fir	2977 (+/- 23)	SF1	2933
			SF2	3012
			SF3	2897
Kendrick	Ponderosa equivalent	2426 (+/- 7)	Ken1A	2426
			Ken1B	2427
			Ken1C	2406
	Mixed Conifer equivalent	2606 (+/- 22)	Ken2A	2640
			Ken2B	2566
			Ken2C	2612
	Spruce-fir equivalent	2984 (+/- 20)	Ken3A	2980
			Ken3B	3021
			Ken3C	2951

The stand-replacing Pumpkin fire in 2000 on the nearby Kendrick Mountain (35.412, -111.876) provided a non-forested elevational gradient control and allowed for an additional test of forest canopy cover, since Kendrick represented natural tree removal habitats, with virtually no trees along the entire gradient, compared to the later succession habitats found on the San Francisco Peaks. Nine sites, at each San Francisco Peaks equivalent elevations, were set up on Kendrick Mountain (Table 1). We compared pollinator communities along these similar elevational gradients between the San Francisco Peaks (unburned, up to 88% canopy cover) and Kendrick Mountain (15 years post burn), where over 99% of trees were naturally removed by the 2000 Pumpkin fire started by lightning [38]. The centroid distance of study areas between the two mountains was 15 kilometers, reducing the potential complication of geographic variance. The study gradients for both mountains included three elevations on Kendrick and the San Francisco Peaks. A total of nine meadow sites from Kendrick Mountain were matched for elevation with the nine sites from the San Francisco Peaks; because there was no forest on Kendrick Mountain we only established one array at each site in a meadow habitat. The average distance between sites on Kendrick Mountain was 0.46 +/- 0.12 kilometers.

### Sampling methods

At each site, the basic sampling unit consisted of one pollinator cup array, which is similar to a pan trap that is elevated off the ground [39]. Each array consisted of nine, 12 oz. WestGate colored (unpainted) plastic stadium cups (three white, three fluorescent yellow and three fluorescent blue). White, yellow and blue colors accounted for all of the major flora colors in this area [39]. The outside diameter of the cup opening was 8 centimeters (cm), and the cups were 10.7 cm deep. Each cup was filled with a 50:50 mixture of water and food-grade propylene glycol (a preservative). The cups were suspended 30 cm above the ground in specially built holders made of polyvinyl chloride (PVC) pipe to approximate the height of most flowering plants [40]. The elevated cups also addressed an issue discussed by Cane, Minckley (40). They found decreased bee diversity samples in pan traps and determined that cups need to be placed at the height level of surrounding flowers [35]. Pollinator cups were set out in two seasons, dry pre-monsoon (June, n = 243) and monsoon (August, n = 243), to capture the comprehensive diversity of the community. Previous sampling effort in this region indicted that the majority of the species were present in any given collection time either in pre-monsoon (May–July) or monsoon (July–September) [35]. Each sampling period consisted of the cups being set up for four full days (i.e. set up morning of day 1 and collected on the morning of day 5). Sampling days were picked based on optimal weather conditions for that season.

A total of 648 cups were placed in arrays during a four-year period (2013–2016) on the San Francisco Peaks and 162 cups were placed in arrays during a two-year period (2015–2016) on Kendrick Mountain. All data was analyzed on a per triad (i.e. one cup of each color) basis to account for multiple year sampling and differences in sampling effort on each of the mountains. During the monsoon of 2013, 50% of the pre-monsoon cups were lost to animal damage; in 2014 we switched to a ground pan trap sampling method instead of a cup sampling method in the spruce-fir life zone to address the high animal traffic. No differences were found between pollinator cups and pan samples for species richness or abundance.

### Identification

We identified bees and flies to species, except for challenging bee taxa which were identified at the USDA Bee Biology and Systematics Laboratory in Logan, Utah. Some species are unresolved at this time, and in these cases, we assigned “morphospecies” designations. Reference collections are stored at Northern Arizona University and all reference species have been digitally cataloged in the Symbiota Collections of Arthropod Network (SCAN) online data portal.

## Statistical analysis

### Elevation effects

To test whether bee abundance, fly abundance, bee richness, and fly richness changed along the elevational gradient and between meadow and forest habitats we ran two generalized liner mixed effect models (GLMM). We examined abundance and species richness as our response variables. For both models, taxa, elevation, and habitat were treated as fixed effects; year, season, and site were treated as random effects. The random effect season was nested within years with a cross random effect of site, as we were primarily interested in the effects of life zone and habitat not year, season, and site. We ran this data with and without the interaction between taxa and elevation. Our data was count data, so we ran each model with Poisson distribution and negative binomial distribution; AIC (Akaike information criterion) showed greater fit for the Poisson distribution model (S3 Table). GLMM analysis was performed using R.3.1.2 and R packages lme4 [41] and arm [42].

We performed a non-metric multidimensional scaling ordination (NMDS) using a Bray-Curtis distance matrix to visualize differences in community composition along the elevational gradient and among habitat types (i.e. meadow and forests), followed by a post hoc Multiple Response Permutation Procedure (MRPP) to test for significance. All analyses were done using R.3.1.2 [43] and packages vegan [44] and ecodist [45]. Additionally, we performed a SIMPER analysis to identify the species that were driving the most dissimilarity between elevations. The SIMPER analysis was conducted using R.3.1.2 [43] and the vegan [44] package. We also ran an indicator species analysis to test which species are indictors between of the three elevations. The indicator species analysis was conducted in R.3.1.2 using the labdsv [46] and indicspecies [47] packages.

### Canopy cover effects

In order to understand the effects of meadow availability on pollinators, we quantified tree canopy cover at all elevations. Images were taken from Google Earth 5.1.3533.1731 [48] at a 100-meter resolution for each of the 18 total sites (9 from the San Francisco Peaks and 9 from Kendrick Mountain). The images were then uploaded in ImageJ 1.46r [49]. To measure the percent canopy cover we took the google images, converted them to black and white, and measured the amount of tree canopy cover versus ground cover/bare ground. Canopy cover measurements were conducted following the methods of Campillo et al. [50].

We also wanted to understand how tree canopy cover correlated with the abundance of the flowering plant communities (quantified by abundance and richness counts along three 60 meter x 1 meter belt transect at each site), bee abundance, fly abundance, bee species richness, and fly species richness. To test these relationships, we ran five linear regressions comparing canopy cover to each of the five variables: plant cover, bee abundance and fly abundance, bee richness, and fly richness. Regression analysis was performed using base R 3.1.2.

In addition to our ordination analysis we conducted two analyses of species importance between habitats. We conducted a SIMPER analysis which indicated what species were the most dissimilar between meadow and forest habitats in the Bray-Curtis distance matrix. We performed this analysis for all three elevations. The SIMPER analysis was conducted using R.3.1.2 [43] and the vegan [44] package. Additionally, we ran an indicator species analysis to test which species are indictors between meadow and forest habitats at each of the three elevations. The indicator species analysis was conducted in R.3.1.2 using the labdsv [46] and indicspecies [47] packages.

## Results

### Changes in abundance and species richness along elevational gradients

Over the four-year period (2013–2016) 11,171 individuals were collected, representing 128 bee species (S1 Table) and 96 fly species (Ss Table). 8,640 individuals were collected in cups along the San Francisco Peaks (2013–2016), 1,512 individuals were collected from Kendrick Mountain (2015–2016), and 1,019 were collected off flowering plants on the San Francisco Peaks in 2016. The San Francisco Peaks had a total of 90 fly species and 101 bee and Kendrick Mountain had 32 fly species and 61 bee species. Collection from pollinator cups included other flower-visiting insects, such as butterflies, beetles and true bugs. These insects were excluded from our analysis as 1) they made up less than 10% of our total insect abundance and 2) their abundance did not change along the elevational gradient.

### Elevation effects

**Fig 1:**
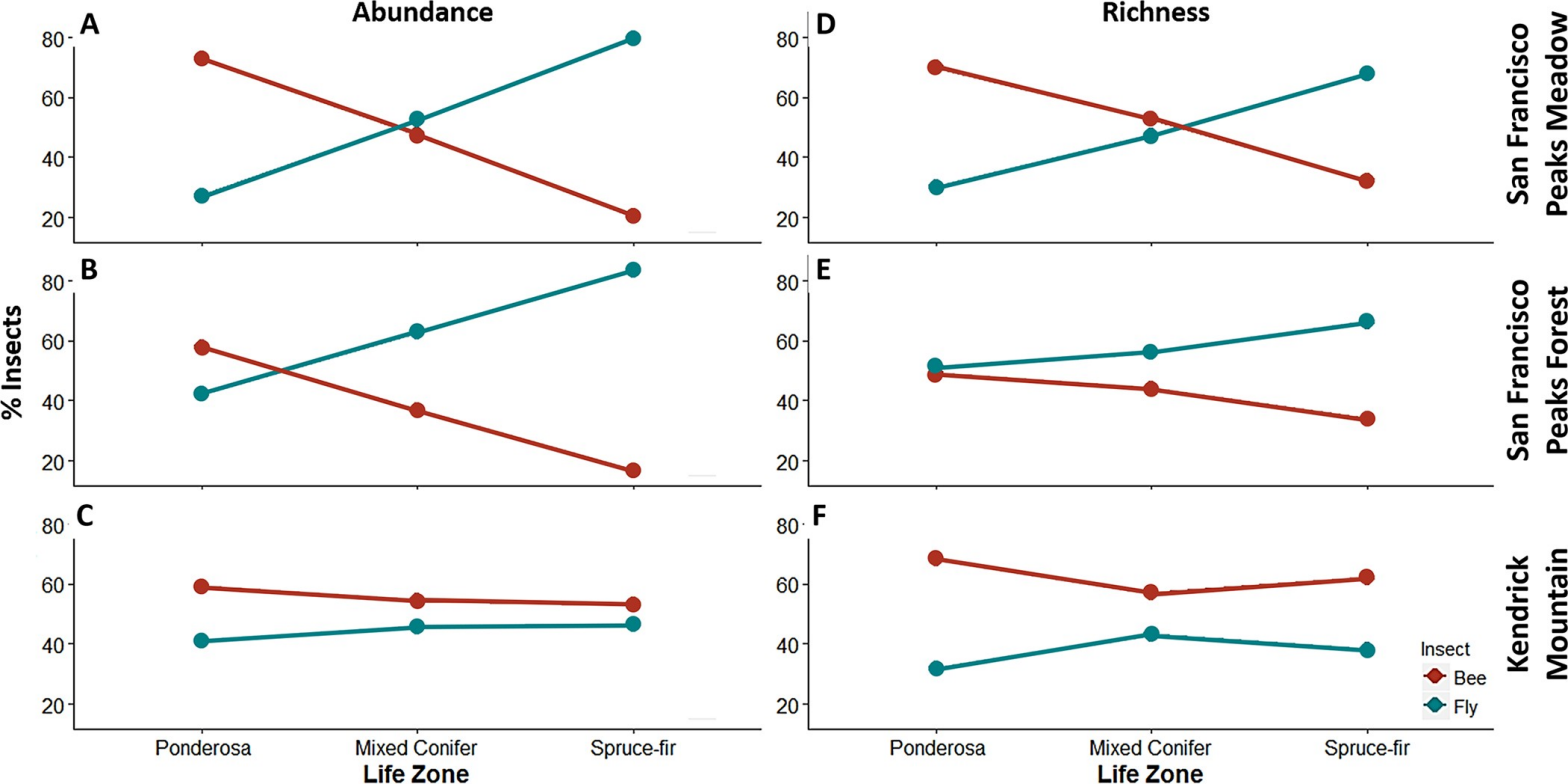
Cup sampling shows changes in both relative abundance and species richness for bees and flies along elevational gradients on two mountains. Changes in abundance of San Francisco Peaks meadow (A) and forest (B) as well as Kendrick Mountain, a no canopy cover mountain (C). Changes in richness of San Francisco Peaks meadow (D) and forest (E) as well as Kendrick Mountain, a no canopy cover mountain (F). Bee species are depicted in as the red line and fly species are depicted as the blue lines. Mean and Standard Errors displayed.

The transition from bee-to-fly dominated communities along an elevational gradient on the San Francisco Peaks was supported both in relative abundance (Z = 118.58, p < 0.001, Fig 1, S4 Table) and relative species richness (Z = 122.47, p < 0.001, Fig 1, S4 Table) data for all years; in both instances (abundance and richness) bees decreased as elevation increased and flies increased as elevation increased. Each year over a four-year period, bees decreased in abundance among the three life zones as elevation increased; from 65% at the ponderosa life zone (lowest elevation sampled) to 19% at spruce-fir (highest elevation sampled) (Z = -5.605, p < 0.001). Bee species richness also declined along the elevational gradient from 64% at ponderosa to 33% at spruce-fir (Z = -4.007, p < 0.001Fig 1). Conversely, flies increased along the elevational gradient in relative species richness (35% at ponderosa to 81% at spruce-fir) and abundance (36% to 67% respectively). At mixed conifer, bees and flies were not statistically different between relative bee and fly abundance and species richness (abundance: 49% bees and 51% flies (p = 0.296), species richness: 48% bees, 52% flies, (p = 0.326).

**Fig 2:**
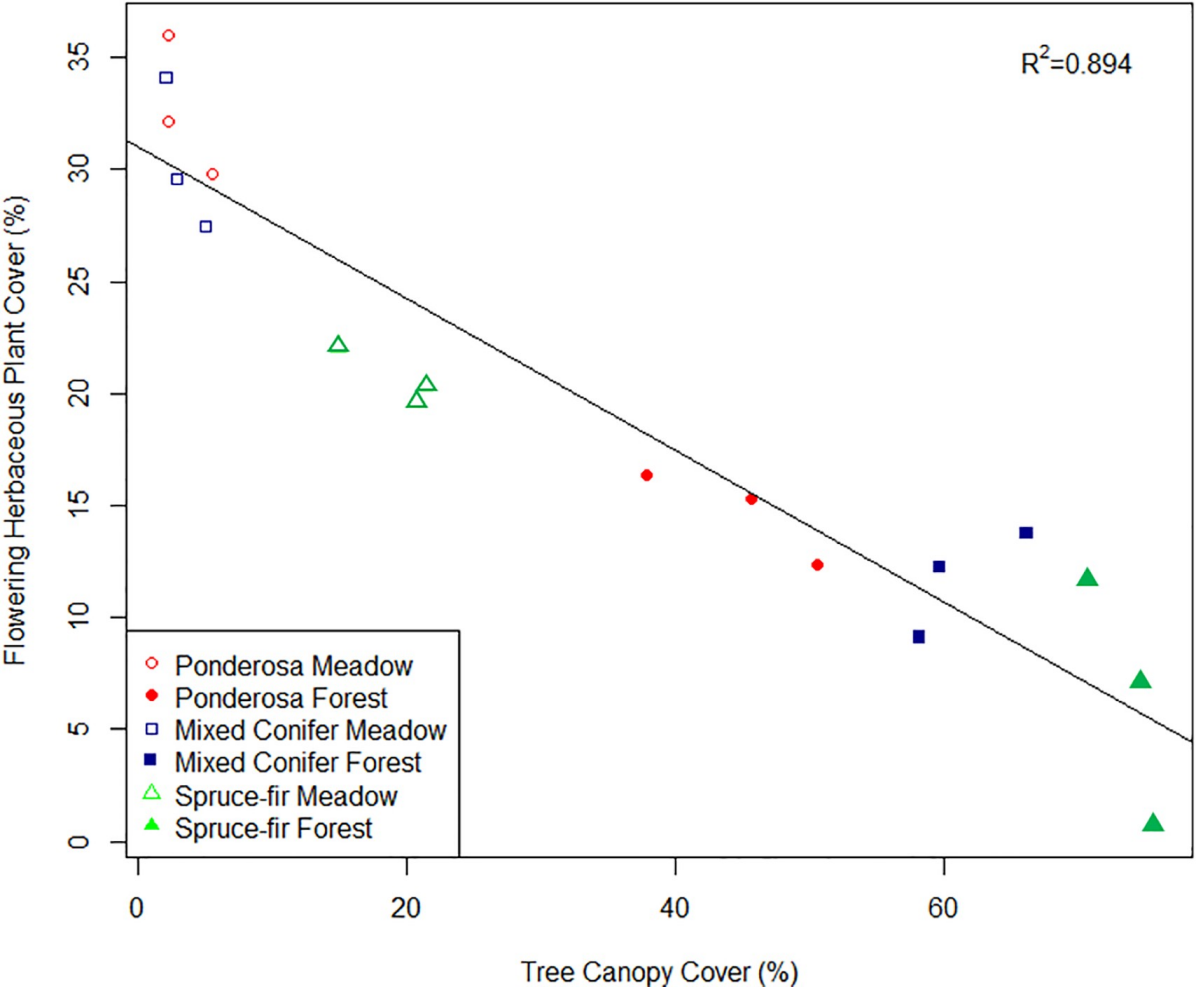
Change in flowering herbaceous plant cover relative to tree canopy cover in meadow (closed symbols) and forest habitats (open symbols). Symbols denote life zones means. As canopy cover increases flowering plant cover decreases (*f =* 135.90 *p* < 0.001, R^2^ = 0.894). While there is a range in floral resource at a low canopy cover, as canopy cover increases the amount of flowering plants becomes limited.

As canopy cover increased, bee abundance decreased (p < 0.001, R^2^ = 0.784, S1 Fig) and fly abundance increased (p < 0.001, R^2^ = 0.811, S1 Fig). Likewise, the same trend is seen with species richness; as canopy cover increased bee species richness decreased (*R*^*2*^ = 0.8228, p < 0.001), and fly species richness increased (*R*^*2*^ = 0.8573, *p* < 0.001). Tree canopy cover was negatively correlated with the amount of flowering herbaceous plants. As canopy cover increased, flowering herbaceous plants decreased (Fig 2, p < 0.001, R^2^ = 0.8947).

**Fig 3:**
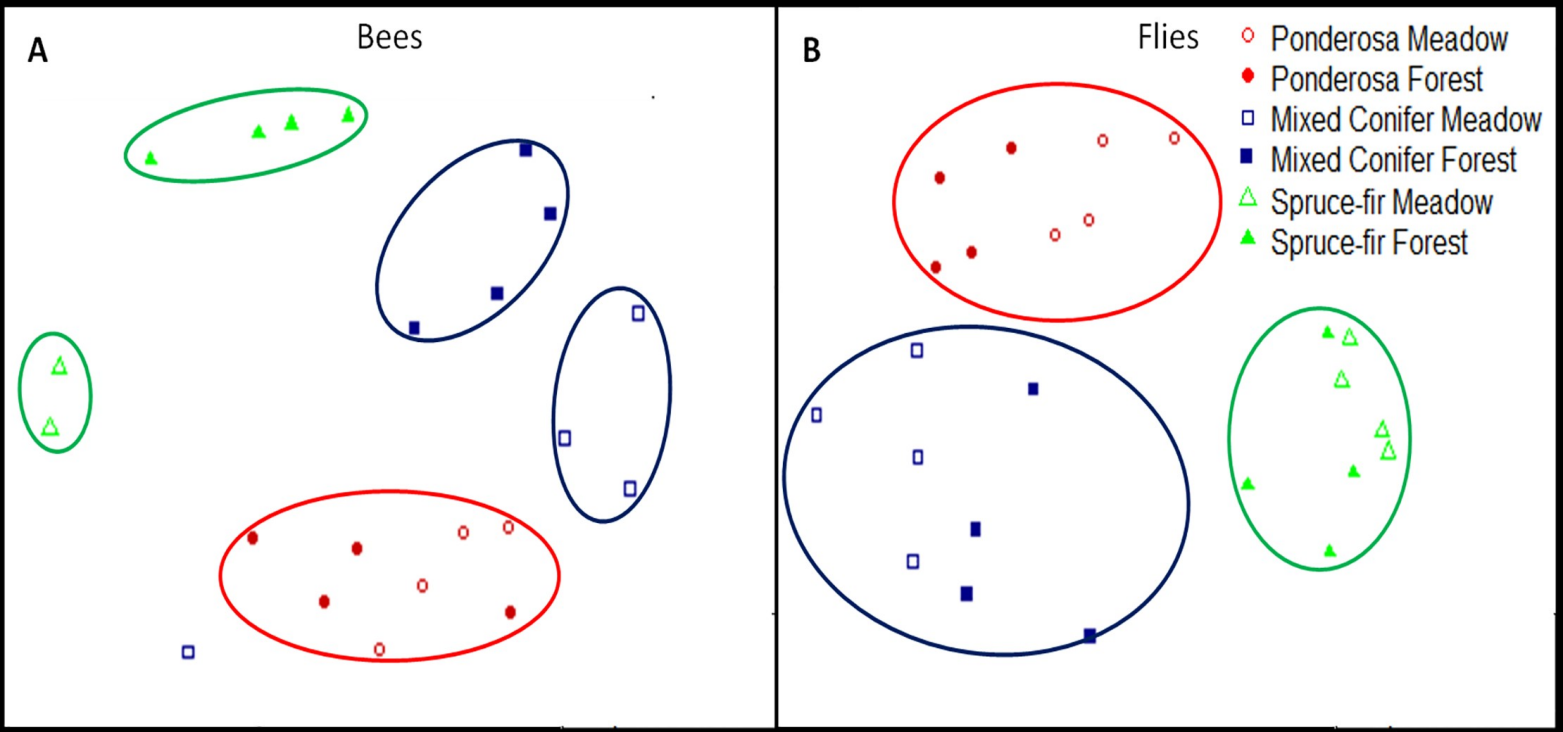
Non-metric multidimensional scaling ordination (NMDS) comparison of bees (A) and flies (B) between forest versus meadow habitats at each of the three elevation vegetation types. Bees (A): all elevations were distinct (p < 0.05) from one another, but meadow and forest habitats were not different in community composition between the three elevation zones; ponderosa (p = 0.0587), mixed conifer (p = 0.1148) and spruce-fir (p = 0.0778). Flies (B): Fly community composition was distinct among the three elevation zones (ponderosa (p = 0.0435), mixed conifer (p = 0.0.030) and spruce-fir (p < 0.0001)), but not between forest and meadow habitat (p < 0.05). Ellipses in graph represent non-significant point groupings (i.e. paired MRPPS between points were less than 0.05).

Community composition was also different among all life zones for both bees and flies. Bee and fly communities were statistically different among all life zones (MRPP: p < 0.0001 (bees), p < 0.0001(flies), Fig 3). SIMPER analysis revealed eight species that contributed the most dissimilarity between ponderosa and mixed conifer and seven species that contributed the most to the dissimilarity between mixed conifer and spruce-fir (S5 Table). Indicator species analysis identified *Perdita* sp., *Halictus* sp. and *Andrena crinita* as indicators between ponderosa and mixed conifer and only *Osmia juxta* for indicator species between mixed conifer and spruce-fir (S6 Table).

### Canopy cover effects

Our hypothesis that community composition along an unburned mountain would show greater differences in meadow and forest habitats was supported with bees, but not flies. Community composition along the San Francisco Peaks between habitats at any of the life zones differed for bees but did not differ for flies (Fig 3). For bees, spruce-fir meadow and forest and mixed conifer meadow and forest pollinator communities differed between habitats (*p =* 0.022 (spruce-fir), *p =* 0.030 (mixed conifer); however, ponderosa meadow and forest habitats did not differ (*p =* 0.085). In contrast, the fly community composition of meadow and forest canopies was not significantly different. No fly community composition at any life zone differed between meadow and forest habitat (ponderosa (*p =* 0.054), mixed conifer (*p =* 0.079) nor spruce-fir (*p =* 0.117)).

SIMPER analysis for bees between sites showed thirteen bee species in ponderosa, nine bee species in mixed conifer and six bee species in spruce-fir that contributed to 70% of the dissimilarity matrix between meadow and forest habitats (S5 Table). Indicator species identified *Protodufourea* sp. as an indicator of ponderosa meadow and forest, *Andrena crinita* for mixed conifer meadow and forest and *Bombus ferridus*, *Bombus appositus*, and *Osmia juxta* in spruce-fir meadow and forest (S6 Table).

Additionally, our hypothesis that the transition from bees-to-flies along a burned mountain (i.e. where little to no canopy cover existed) would not persist was also supported. The shift in pollinator taxa did not take place along the burned Kendrick mountain in either relative abundance (F = 2.413, p = 0.141) or species richness (F = 1.349, p = 0.264). Additionally, fly abundance on the unburned mountain was 3x greater than on our burned mountain (F = 1.962, p = 0.034).

## Discussion

### Bee-to-fly transition as elevation increases

Our research provides comprehensive support for the bee-to-fly transition hypothesis, which predicts that bees dominate communities at lower life zone environments and are replaced by flies at higher elevations [2, 3, 51]. We show that both abundance and species richness indicated a clear pattern of bee dominated communities at the low elevation life zone (ponderosa) and fly dominated communities at the high elevation life zone (spruce-fir), with an equal abundance and species richness of flies to bees at our mid-elevation life zone (mixed conifer). Additionally, we found that canopy cover along elevational gradients may be a driving factor in determining pollinator community composition.

On the San Francisco Peaks, the rapid drop in bees above mixed conifer is likely due to three factors: increased tree canopy cover, increased precipitation, and decreased temperature. In this study we primarily addressed the increase in tree canopy cover and subsequent decrease in floral resources due to the increase in canopy cover [52]; which we presume has a much larger impact on bees than it does flies due to the strong relationship that bees have with their host plants. An increase in canopy cover can also have indirect effects on temperature such as increased humidity. A survey of flower visitors in South America along a precipitation gradient supports the notion that bees are more abundant than flies in low precipitation and low humidity areas, whereas flies are more abundant in areas that have high humidity and high amounts of precipitation [53]. Most bees nest in microhabitats characterized by dry soils and warm temperature [54]. Temperature is also important for adult bees, because a specific ambient body temperature is required to forage for resources [55]. An increase in canopy cover limits the meadow habitats, thus decreasing floral resources, which impacts bees more than flies. However, it is difficult to separate temperature and precipitation from canopy cover effects alone.

We suggest that flies are relatively more abundant and diverse in spruce-fir, because open habitats, in lower elevations, which are used for both nesting and floral resources are reduced for bees at higher elevations, leading to flies becoming relatively more abundant and diverse above mixed conifer. Higher-elevation life zones may also be more conducive for flies due to forested wetter habitats that generally provide more resources for dipteran offspring [56] including parasite-host interactions. Unlike bees, flies do not require as much nectar and pollen and are therefore less limited by decreased floral resources at higher elevations [57].

The ecology of dipteran larvae is poorly known, including basic information on substrates (e.g., soil, carrion) and feeding type (e.g., parasitoid, scavenger). Species-level accounts covering larval ecology are only known for a few genera [58] and in many cases only one species in a genus has been studied. Of the 96 species of flies in our study only 3% have larval biology known in terms of food and substrate. Although, we are confident that we can ascribe at least larval feeding modes for 90% of the species, due to the generality of the feeding modes found in a single family or genus. The most diverse and abundant taxa in our study were Tachinidae, which are insect parasites, primarily attacking Lepidoptera and Coleoptera. Together, with other parasites and predators they constituted 70% of species larval feeding type. The rest of larval feeding types included scavengers at 21% and herbivores 9%. Regardless of feeding type, 46% of the fly species likely live in soil or carrion. All these modes of feeding are typically enhanced in areas of higher precipitation and humidity, such as high elevation areas along mountain gradients.

The SIMPER analysis showed the dissimilarity between ponderosa and mixed conifer was primarily a mix of species from all five bee families, likely due to change in flora host or temperature. However, the dissimilarity from mixed conifer to spruce-fir was due to members *Bombus* and Megachilidae suggesting that Megachilidae species and *Bombus* species are more important at higher elevations. This is likely due to increased wood nesting substrates for Megachilidae [59] and *Bombus* being adapted to colder environments[60].

### Tree canopy cover effects

On our unburned mountain sites (San Francisco Peaks) the transition zone, where bees and flies were equally abundant, occurred at the mixed conifer life zone, about 2,600 meters. However, the transition zone on our 15-year-old burned site (Kendrick Mountain) occurred higher up at about 3,500 meters, in the spruce-fir equivalent life zone, and never switched to a fly dominated pollinator community. At our burned site the floral cover was five times greater than at the equivalent life zone at our unburned site, suggesting that this lack of changing dominate pollinator taxa could be due to the limitation of floral resources in high elevation and high canopy covered areas. The burned areas consist of continuous meadows that produce more floral resources that are likely able to support a bee dominated pollinator community. However, these changes may be driven largely by succession. Early succession burned areas tend to produce more flowering plants than later successional plots, which has been known to increase bee species richness in early successional environments [61].

Additionally, we expect that increasing tree canopy cover creates microclimates that are more conducive to fly larval development compared to bees [62]. Increasing forest canopy cover also decreases meadow sizes and resources, creating a patchy habitat of small, distant meadows at our highest elevation. We did not find that ponderosa meadow and forest habitats were distinctly different, presumably due to the large meadows and smaller, less dense forest habitats. Fly communities were not distinctly different, most likely because many flies rely on both meadow and forest habitats for their lifecycle development. Although, bee communities were different between meadows and forests habitats, community composition was more similar to its partner habitat than the same habitat at a different life zone (i.e, ponderosa meadow was more similar to ponderosa forest than mixed conifer meadow). However, fly communities were more closely related on an elevation/life zone level than a habitat level. Vazquez and Simberloff (63) found similar patterns when they examined pollinator communities under tree canopy cover only[63]. They found that fly diversity was greater than bee diversity in the forest and the composition of the communities did not change throughout; [64]. This study supports our conclusion that canopy cover is an ecological limitation for bees at higher elevations.

The SIMPER analysis identified the species driving the dissimilarity between meadow and forest habitats along the gradient. With an increase in elevation there were fewer species that contributed to 70% of the dissimilarity, therefore each species was more important in their contribution to dissimilarity. This is what we expected because the distinction between forest and meadows was more diffuse in ponderosa, with no pure meadows and grassland habitat continuous through forested areas. Meadows and forests became increasingly distinct as elevation increased.

The two primary characteristics of the species identified by the SIMPER analysis at all elevations/life zones were meadow specialists (i.e. they only occurred in meadow habitats) and very common species at their respective life zones.

Indicator analysis showed only six species as indicators along the gradient. The morphospecies, Protodufourea001, was the only indicator species at the ponderosa life zone and had a strong indication for the forest habitats. The indicator species for mixed conifer were *Andrena crinita* and Perdita003, both of which were indicator species for meadow habitats. *Bombus ferridus*, *Bombus appositus* and *Osmia juxta* were indicator species at spruce-fir. *B*. *ferridus* and *B*. *appositus* were indicators for the meadow habitat. *O*. *juxta* was an indicator for the forest habitat, likely due to their strong ties to wood nesting resources.

We suggest that increasing canopy cover primarily limits bees due to the subsequent decrease in flowering plants. Farwig, Bailey (65) reported a significant decrease of bee pollinator activity in isolated meadows surrounded by dense canopy cover. In open meadows and sparse canopy cover, forests had no effect on bee pollinator productivity [65]. Bee richness was also found to be limited by an increase in canopy cover along an open-forest gradient [66]. These results are congruent with what was found in this study where the ponderosa life zone, with sparse canopy cover, showed no differences between habitat, while the two denser life zones, mixed conifer and spruce-fir, showed a specialization of bees on habitats.

Unlike most holometabolous insects, where the larval stage feeds on a different resource than the adult, both the larval stage and the adult stage of bees feed on the same pollen and nectar resources [67]. This is an additional limitation on bees, where in areas of decreased floral resources, female bees not only have to find food for themselves, but their offspring as well, decreasing the likelihood of offspring produced [68]. Flies on the other hand do not have the same resource requirements during all life stages and therefore can survive in a more limited resource environment [69].

While we believe that canopy cover is an important limiting factor for bees below tree line, this cannot explain the lack of bees above tree line, where no canopy cover exists. Although our data is still anecdotal, it supports the notion that flies continue to dominate pollinator communities over bees at high elevations [7]. Other factors such as temperature and precipitation are most likely to explain this pattern.

High elevation communities, and tree species in particular, are predicted to be highly susceptible to climate change [70], scores of studies in the southwestern United States have already documented widespread tree mortality and this phenomena is global in scope [71, 72]The existence of tree habitat is widely known to be threatened by climate change [70, 72]. However, to our knowledge there is no literature that makes the same claims for meadow or grassland habitats, although community composition of meadows may be significantly disrupted. We predict the bee-to-fly transition will shift upward with increasing temperatures and loss of forest habitat on mountains in the southwestern United States. Our results suggest that a subsequent increase in open meadows should benefit bees more than flies.

## Conclusion

Along elevational gradients bees have been shown to dominate pollinator communities at lower elevations. However, flies persist along elevational gradients and show an increase in abundance and species richness at the highest elevations. We propose that an important driver of this transition is an increase in canopy cover as elevation increases, likely reducing resources for bee species and increasing larval resources for fly species. Our results further support the notion that this bee-to-fly transition will shift up in elevation with the removal of trees.

## Supporting information

S1 Table Species list of all bees collected though out the elevation gradient. MCM = mixed conifer meadow, MC = mixed conifer, PP = ponderosa pine, SF = spruce-fir.
(DOCX)

S2 Table Species list of all flies collected though out the elevation gradient. MCM = mixed conifer meadow, MC = mixed conifer, PP = ponderosa pine, SF = spruce-fir.
(DOCX)

S3 Table GLMM model selection showing the two model, model selection approach. All models included the random effect; only the predictor variables included in each model are shown.
(DOCX)

S4 Table **A:** GLMM table showing relative bee abundance & species richness and relative fly abundance & species richness. Comparison includes life zone, habitat, and year, as well as their interaction terms. **B:** ANOVA tables for the Kendrick study showing relative bee abundance & species richness and relative fly abundance & species richness, and comparison in insect abundance between the two elevational gradients **C**. ANOVA table to show no differences between sampling methods of collecting pollinators from cup traps versus collecting directly off flowers.
(DOCX)

S5 Table Percentage contribution by the top 70% of the Bray-Curtis dissimilarity matrix for differences between habitat at the three life zones (PPM = ponderosa meadow, PPF = ponderosa forest, MCM = mixed conifer meadow, MCF = mixed conifer forest, SFM = spruce-fir meadow, SFF = spruce-fir forest, PP = Ponderosa, MC = Mixed Conifer, SF = Spruce-fir). (DOCX)

S6 Table Indicator values (IV) and related significance between meadow and forest habitat type at the three life zones (PPM = ponderosa meadow, PPF = ponderosa forest, MCM = mixed conifer meadow, MCF = mixed conifer forest, SFM = spruce-fir meadow, SFF = spruce-fir forest). (DOCX)

S1 Fig Differences in bee abundance (A) and fly abundance (B) and bee species richness (C) and fly species richness (D) among life zones and between habitats based on average numbers per cup. Bee and fly abundance and species richness among life zones for forest and meadow habitats.
(TIFF)

S2 Fig Bee and fly abundance and species richness among life zones on Kendrick Mountain. Differences in bee and fly abundance (A) and bee and fly species richness (B) among life zones and between habitats based on average numbers per cup.
(TIFF)
