# Supplementary material for: The transition from bee-to-fly dominated communities with increasing elevation and greater forest canopy cover
Source: PLoS One. 2019 Jun 12;14(6):e0217198. doi: 10.1371/journal.pone.0217198 (PMC6561536; doi:10.1371/journal.pone.0217198)
Supplement: S1 Table — MCM = mixed conifer meadow, MC = mixed conifer, PP = ponderosa pine, SF = spruce-fir. (DOCX) [file pone.0217198.s001.docx]

**S1 Table**: Species list of all bees collected though out the elevation gradient. MCM= mixed conifer meadow, MC= mixed conifer, PP= ponderosa pine, SF= spruce-fir.

| **Family** | **Genus** | **Species** | **PP** | **MC** | **SF** |
| --- | --- | --- | --- | --- | --- |
| Andrenidae | Andrena | algida |  | 1 |  |
|  | Andrena | argemonis | 1 | 1 |  |
|  | Andrena | belandrena n. sp. | 1 |  |  |
|  | Andrena | brevipalpis |  | 1 |  |
|  | Andrena | commoda |  | 1 |  |
|  | Andrena | crataegi |  | 1 |  |
|  | Andrena | crinita |  | 1 |  |
|  | Andrena | mariae | 1 | 1 |  |
|  | Andrena | medionitens |  | 1 |  |
|  | Andrena | miranda | 1 | 1 |  |
|  | Andrena | nubecula | 1 | 1 | 1 |
|  | Andrena | nuda |  | 1 |  |
|  | Andrena | perpunctata |  | 1 |  |
|  | Andrena | platyrhina |  | 1 |  |
|  | Andrena | striatifrons |  | 1 |  |
|  | Calliopsis | callops |  | 1 |  |
|  | Calliopsis | zebrata | 1 |  |  |
|  | Pseudopanurgus | albitarsis |  |  | 1 |
|  | Pseudopanurgus | atricornis |  |  | 1 |
|  | Pseudopanurgus | boylei |  | 1 | 1 |
|  | Pseudopanurgus | illustris |  | 1 | 1 |
|  | Pseudopanurgus | porterae |  | 1 |  |
| Apidae | Anthophora | affabilis | 1 |  |  |
|  | Anthophora | californica | 1 |  |  |
|  | Anthophora | exigua | 1 |  |  |
|  | Anthophora | furcata | 1 |  |  |
|  | Anthophora | marginata |  |  | 1 |
|  | Anthophora | montana | 1 | 1 |  |
|  | Anthophora | petrophila | 1 | 1 | 1 |
|  | Anthophora | terminalis | 1 | 1 | 1 |
|  | Anthophora | urbana | 1 | 1 | 1 |
|  | Anthophora | ursina | 1 |  |  |
|  | Apis | mellifera | 1 | 1 |  |
|  | Bombus | huntii | 1 | 1 | 1 |
|  | Bombus | morrisoni | 1 | 1 |  |
|  | Bombus | nevadensis | 1 | 1 |  |
|  | Bombus | occidentalis |  | 1 | 1 |
|  | Centris | rhodopus | 1 |  |  |
|  | Ceratina | nanula | 1 |  |  |
|  | Ceratina | neomexicana | 1 | 1 |  |
|  | Ceratina | pacifica | 1 |  |  |
|  | Diadasia | australis | 1 | 1 |  |
|  | Diadasia | diminuta | 1 | 1 |  |
|  | Diadasia | rinconis | 1 | 1 |  |
|  | Epeolus | compactus |  | 1 |  |
|  | Epeolus | flavofasciatus |  | 1 |  |
|  | Ericrocis | lata | 1 | 1 |  |
|  | Eucera | ternitella | 1 |  |  |
|  | Melissodes | agilis | 1 |  |  |
|  | Melissodes | coloradensis | 1 | 1 |  |
|  | Melissodes | confusa | 1 |  |  |
|  | Melissodes | gilensis | 1 | 1 |  |
|  | Melissodes | glenwoodensis | 1 |  |  |
|  | Melissodes | grindeliae | 1 | 1 |  |
|  | Melissodes | pallidisignata | 1 | 1 |  |
|  | Melissodes | paroselae | 1 | 1 |  |
|  | Melissodes | perpolitus | 1 | 1 |  |
|  | Melissodes | rivalis | 1 | 1 |  |
|  | Melissodes | rustica | 1 | 1 |  |
|  | Melissodes | tristis | 1 | 1 |  |
|  | Nomada | texana |  | 1 |  |
|  | Nomada | utahensis |  | 1 |  |
|  | Nomada | zebrata | 1 |  |  |
|  | Nomia | foxii | 1 | 1 |  |
|  | Nomia | tetrazonata | 1 |  |  |
|  | svastra | sp1 | 1 |  |  |
|  | Triepeolus | sp1 |  | 1 |  |
|  | Xeromelecta | californica |  | 1 |  |
| Colletidae | Colletes | compactus | 1 |  |  |
|  | Colletes | gilensis | 1 |  |  |
|  | Colletes | kincaidii | 1 | 1 |  |
|  | Colletes | paniscus |  | 1 |  |
|  | Hylaeus | annulatus |  | 1 |  |
|  | Hylaeus | wootoni | 1 |  |  |
| Halictidae | Agapostemon | angelicus | 1 | 1 | 1 |
|  | Agapostemon | texanus | 1 | 1 |  |
|  | Halictus | ligatus |  | 1 | 1 |
|  | Halictus | tripartitus |  |  | 1 |
|  | Lasioglossium | hasioglassum | 1 | 1 |  |
|  | Lasioglossum | desertum | 1 | 1 | 1 |
|  | Lasioglossum | egregium | 1 | 1 |  |
|  | Lasioglossum | sysimbrii | 1 | 1 |  |
|  | Lasioglossum | trizonatum | 1 | 1 |  |
|  | Nomia | tetrazonata |  | 1 | 1 |
|  | Sphecodes | 1 |  | 1 |  |
|  | Sphecodes | 2 |  | 1 |  |
|  | Sphecodes | 3 |  | 1 |  |
| Megachilidae | Anthidium | clypeodentatum |  | 1 |  |
|  | Anthidium | cylpeodeutatea |  | 1 |  |
|  | Anthidium | illustre | 1 | 1 |  |
|  | Anthidium | mormonum | 1 |  |  |
|  | Anthidium | porterae | 1 |  |  |
|  | Anthidium | rudbeckiarium | 1 | 1 |  |
|  | Ashmeadiella | bucconis |  | 1 |  |
|  | Ashmeadiella | cactorum | 1 |  |  |
|  | Coelioxys | apacheorum | 1 |  |  |
|  | Coelioxys | erysimi | 1 |  |  |
|  | Coelioxys | gilensis | 1 |  |  |
|  | Coelioxys | moesta | 1 | 1 |  |
|  | Dianthidium | cressonii | 1 | 1 |  |
|  | Dianthidium | heterulkei | 1 | 1 |  |
|  | Dianthidium | ulkei | 1 |  |  |
|  | Hoplitis | grinnelli | 1 |  |  |
|  | Lithurgus | apicalis | 1 | 1 |  |
|  | Megachile | comata | 1 |  |  |
|  | Megachile | fidelis | 1 | 1 |  |
|  | Megachile | fortis | 1 |  | 1 |
|  | Megachile | frigida | 1 | 1 | 1 |
|  | Megachile | manifesta | 1 | 1 |  |
|  | Megachile | melanophaea |  | 1 | 1 |
|  | Megachile | mellitarsis | 1 | 1 |  |
|  | Megachile | montivaga | 1 | 1 |  |
|  | Megachile | mucida | 1 |  | 1 |
|  | Megachile | perihirta | 1 |  |  |
|  | Megachile | policaris | 1 | 1 |  |
|  | Megachile | subexilis | 1 | 1 |  |
|  | Megachile | sublaurita | 1 |  |  |
|  | Osmia | albolateralis |  |  | 1 |
|  | Osmia | bucephala | 1 | 1 |  |
|  | Osmia | coloradensis | 1 | 1 | 1 |
|  | Osmia | juxta | 1 | 1 | 1 |
|  | Osmia | lignaria |  | 1 |  |
|  | Osmia | montana |  |  | 1 |
|  | Osmia | simillima |  | 1 | 1 |
|  | Osmia | subaustralis |  |  | 1 |
|  | Osmia | texana | 1 | 1 | 1 |
|  | Paranthidium | jugatorium | 1 |  | 1 |
|  | Stelis | rudbeckiarum |  | 1 | 1 |
