# Supplementary material for: The transition from bee-to-fly dominated communities with increasing elevation and greater forest canopy cover
Source: PLoS One. 2019 Jun 12;14(6):e0217198. doi: 10.1371/journal.pone.0217198 (PMC6561536; doi:10.1371/journal.pone.0217198)
Supplement: S2 Table — MCM = mixed conifer meadow, MC = mixed conifer, PP = ponderosa pine, SF = spruce-fir. (DOCX) [file pone.0217198.s002.docx]

**S2 Table**: Species list of all flies collected though out the elevation gradient. MCM= mixed conifer meadow, MC= mixed conifer, PP= ponderosa pine, SF= spruce-fir.

| **Family** | **Genus** | **Species** | **PPM** | **MCM** | **SFM** |
| --- | --- | --- | --- | --- | --- |
| Anthomyiidae | Anthomyiid | 1 | 0 | 1 | 0 |
|  | Anthomyiid | 2 | 1 | 1 | 0 |
|  | Anthomyiid | 3 | 1 | 1 | 0 |
| Bombyliidae | Bombyliidae | 1 | 0 | 0 | 1 |
|  | Bombyliidae | 2 | 0 | 0 | 1 |
|  | Bombyliidae | 3 | 0 | 1 | 1 |
|  | *Bombylius* | major | 0 | 0 | 1 |
|  | *Conophorus* | fenestratus | 0 | 1 | 1 |
|  | *Hemipenthes* | sinuosa | 1 | 1 | 1 |
| Calliphoridae | *Calliphoridae* | 1 | 0 | 1 | 0 |
|  | *Chysomya* | megacephala | 0 | 1 | 1 |
|  | *Cynomya* | cadaverina | 1 | 1 | 1 |
|  | Cynomya | 1 | 0 | 1 | 0 |
|  | Lucillia | 1 | 0 | 1 | 0 |
| Muscidae | Coenosia | 1 | 0 | 1 | 0 |
|  | Coenosia | 2 | 0 | 1 | 0 |
|  | Coenosia | 3 | 0 | 1 | 0 |
|  | Coenosia | 4 | 1 | 1 | 0 |
|  | Muscid | 1 | 1 | 0 | 0 |
|  | Muscid | 2 | 1 | 0 | 0 |
|  | Muscid | 3 | 1 | 1 | 0 |
|  | Muscid | 4 | 0 | 1 | 1 |
|  | Muscidae | 1 | 0 | 1 | 0 |
|  | Muscidae | 2 | 0 | 1 | 0 |
|  | Muscidae | 3 | 0 | 1 | 0 |
|  | Muscidae | 4 | 1 | 1 | 0 |
|  | Muscidae | 5 | 1 | 1 | 1 |
|  | Muscidae | 6 | 0 | 0 | 1 |
|  | Muscidae | 7 | 0 | 1 | 1 |
| Sarcophagidae | Sarcophaga | 1 | 0 | 1 | 0 |
|  | Sarcophaga | 2 | 0 | 1 | 0 |
|  | Sarcophaga | 3 | 1 | 1 | 0 |
|  | Sarcophagidae | 4 | 0 | 1 | 0 |
| Syrphidae | *Brachyopa* | 1 | 0 | 1 | 0 |
|  | *Chalcosyrophus* | piger | 0 | 1 | 1 |
|  | Chalcosyrphus | 1 | 0 | 1 | 0 |
|  | Chalcosyrphus | 2 | 1 | 1 | 1 |
|  | *Chalcoysrphus* | plesia | 0 | 1 | 1 |
|  | *Chrysotoxum* | integrum | 0 | 1 | 0 |
|  | Chrysotoxum | 1 | 0 | 1 | 1 |
|  | Chrysotoxum | 2 | 0 | 1 | 0 |
|  | Chrysotoxum | 3 | 0 | 1 | 0 |
|  | Chrysotoxum | 4 | 0 | 1 | 0 |
|  | *Eristalinus* | taenipos | 0 | 1 | 1 |
|  | Eristalinus | 2 | 1 | 1 | 0 |
|  | *Eristalis* | obsura | 1 | 1 | 0 |
|  | Eumerus | 1 | 1 | 1 | 0 |
|  | *Eupeodes* | volucris | 0 | 1 | 1 |
|  | *Helophilus* | 1 | 0 | 1 | 1 |
|  | Syrphid | 4 | 1 | 0 | 1 |
|  | Syrphidae | 1 | 0 | 1 | 0 |
|  | Syrphidae | 2 | 0 | 1 | 0 |
|  | Syrphidae | 3 | 0 | 1 | 0 |
|  | Toxomerus | 2 | 0 | 0 | 1 |
|  | Toxomerus | 3 | 0 | 0 | 1 |
|  | Toxomerus | 4 | 0 | 0 | 1 |
|  | Toxomerus | 1 | 0 | 1 | 0 |
|  | *Trichopoda* | pennies | 0 | 0 | 1 |
|  | Trichopoda | 4 | 0 | 1 | 1 |
|  | Trichopoda | 5 | 0 | 1 | 0 |
|  | Villa | 1 | 0 | 0 | 1 |
| Tachinidae | *Adejeania* | vexatrix | 0 | 1 | 1 |
|  | Archytas | 1 | 1 | 1 | 0 |
|  | Archytas | 2 | 1 | 1 | 0 |
|  | Archytas | 3 | 1 | 1 | 1 |
|  | Archytas | 4 | 0 | 1 | 0 |
|  | Archytas | 5 | 0 | 1 | 0 |
|  | Peleteria | 1 | 0 | 1 | 0 |
|  | Peleteria | 2 | 0 | 1 | 0 |
|  | Peleteria | 3 | 0 | 1 | 0 |
|  | *Tachina* | latianulum | 0 | 1 | 1 |
|  | *Tachina* | Protodejeania | 1 | 1 | 1 |
|  | Tachina | 1 | 0 | 1 | 0 |
|  | Tachina | 2 | 0 | 0 | 1 |
|  | Tachina | 3 | 0 | 1 | 0 |
|  | Tachiniade | 1 | 0 | 1 | 0 |
|  | Tachinid | 4 | 0 | 0 | 1 |
|  | Tachinid | 5 | 0 | 0 | 1 |
|  | Tachinid | 6 | 0 | 0 | 1 |
|  | Tachinidae | 2 | 1 | 1 | 0 |
|  | Tachinidae | 3 | 1 | 0 | 0 |
|  | Tachinidae | 4 | 1 | 0 | 0 |
|  | Tachinidae | 5 | 1 | 0 | 0 |
|  | Tachinidae | 6 | 1 | 1 | 0 |
|  | Tachinidae | 7 | 0 | 1 | 0 |
|  | Tachinidae | 8 | 0 | 1 | 0 |
|  | Tachinidae | 9 | 0 | 0 | 1 |
|  | *Trichopoda* | plumipes | 0 | 1 | 0 |
|  | Trichopoda | 1 | 0 | 1 | 0 |
|  | Trichopoda | 2 | 0 | 0 | 1 |
|  | Trichopoda | 3 | 0 | 0 | 1 |
|  | *Xanthoepalpus* | bicolor | 1 | 1 | 1 |
| Tephritidae | *Euaresta* | bella | 0 | 1 | 0 |
|  | Tephritidae | 2 | 0 | 1 | 0 |
|  | Tephritidae | 3 | 0 | 1 | 0 |
|  | Tephritidae | 1 | 1 | 1 | 0 |
