# Supplementary material for: The transition from bee-to-fly dominated communities with increasing elevation and greater forest canopy cover
Source: PLoS One. 2019 Jun 12;14(6):e0217198. doi: 10.1371/journal.pone.0217198 (PMC6561536; doi:10.1371/journal.pone.0217198)
Supplement: S3 Table — All models included the random effect; only the predictor variables included in each model are shown. (DOCX) [file pone.0217198.s003.docx]

**S3 Table**: GLMM model selection showing the two model, model selection approach. All models included the random effect; only the predictor variables included in each model are shown.

| **Model** | **Family** | **AIC** | **Deviance** | **df.Residuals** |
| --- | --- | --- | --- | --- |
| Abundance ~ Taxa * Life Zone + Habitat | Negative Binomial | 1010.5 | 992.5 | 99 |
| Abundance ~ Taxa + Life Zone + Habitat | Poisson | 1043.3 | 1029.3 | 101 |
| Abundance ~ Taxa * Life Zone + Habitat | Poisson | 1578.3 | 1562.3 | 100 |
| Fly Abundance ~ Life Zone + Habitat | Poisson | 493.1 | 481.1 | 48 |
| Bee Abundance ~ Life Zone + Habitat | Poisson | 485.1 | 473.1 | 48 |
| **Model** | **Family** | **AIC** | **Deviance** | **df.Residuals** |
| Richness ~ Taxa * Life Zone + Habitat | Negative Binomial | 1013.2 | 999.2 | 101 |
| Richness ~ Taxa + Life Zone + Habitat | Poisson | 1267.6 | 1251.6 | 100 |
| Richness ~ Taxa * Life Zone + Habitat | Poisson | 1435.7 | 1423.7 | 102 |
| Fly Richness ~ Life Zone + Habitat | Poisson | 455.6 | 443.6 | 48 |
| Bee Richness ~ Life Zone + Habitat | Poisson | 457.4 | 445.4 | 48 |
| *All models included year and site as random effects, with site nested in year | | | | |
