# Supplementary material for: The transition from bee-to-fly dominated communities with increasing elevation and greater forest canopy cover
Source: PLoS One. 2019 Jun 12;14(6):e0217198. doi: 10.1371/journal.pone.0217198 (PMC6561536; doi:10.1371/journal.pone.0217198)
Supplement: S4 Table — A: GLMM table showing relative bee abundance & species richness and relative fly abundance & species richness. Comparison includes life zone, habitat, and year, as well as their interaction terms. B: ANOVA tables for the Kendrick study showing relative bee abundance & species richness and relative fly abundance & species richness, and comparison in insect abundance between the two elevational gradients C. ANOVA table to show no differences between sampling methods of collecting pollinators from cup traps versus collecting directly off flowers. (DOCX) [file pone.0217198.s004.docx]

**S4 Table: A:** GLMM table showing relative bee abundance & species richness and relative fly abundance & species richness. Comparison includes life zone, habitat, and year, as well as their interaction terms. **B:** ANOVA tables for the Kendrick study showing relative bee abundance & species richness and relative fly abundance & species richness, and comparison in insect abundance between the two elevational gradients **C**. ANOVA table to show no differences between sampling methods of collecting pollinators from cup traps versus collecting directly off flowers.

| **A. Effect of Habitat and Elevation** | | | | | | | | | |
| --- | --- | --- | --- | --- | --- | --- | --- | --- | --- |
| **Abundance** | | | | | **Richness** | | | | |
| **Variables** | Estimate | df | Z -value | P | Variables | Estimate | df | Z -value | P |
| Intercept | 3.741 |  | 118.58 | < 0.001 | Intercept | 3.807 |  | 122.47 | < 0.001 |
| Group | 0.317 |  | 11.52 | < 0.001 | Group | 0.203 |  | 7.442 | < 0.001 |
| **Bee Abundance** | | | | | **Bee Abundance** | | | | |
| **Variables** | Estimate | df | Z -value | P | Variables | Estimate | df | Z -value | P |
| Intercept | 3.534 |  | 16.828 | < 0.001 | Intercept | 3.787 |  | 22.586 | <0.001 |
| Life Zone: Ponderosa | 0.464 |  | 2.403 | 0.016 | Life Zone: Ponderosa | 0.071 |  | 0.662 | 0.508 |
| Life Zone: Spruce-fir | -1.152 |  | -5.605 | < 0.001 | Life Zone: Spruce-fir | -0.441 |  | -4.007 | <0.001 |
| Habitat | -0.231 |  | -2.192 | 0.028 | Habitat | -0.116 |  | -1.661 | 0.096 |
| **Fly Abundance** | | | | | **Fly Abundance** | | | | |
| **Variables** | Estimate | df | Z -value | P | Variables | Estimate | df | Z -value | P |
| Intercept | 4.125 |  | 34.431 | <0.001 | Intercept | 3.973 |  | 31.018 | <0.001 |
| Life Zone: Ponderosa | -0.599 |  | -4.568 | <0.001 | Life Zone: Ponderosa | -0.143 |  | -1.639 | 0.101 |
| Life Zone: Spruce-fir | 0.352 |  | 2.795 | 0.005 | Life Zone: Spruce-fir | 0.263 |  | 3.099 | 0.001 |
| Habitat | 0.249 |  | 1.48 | 0.138 | Habitat | 0.104 |  | 1.185 | 0.236 |
