# Supplementary material for: The transition from bee-to-fly dominated communities with increasing elevation and greater forest canopy cover
Source: PLoS One. 2019 Jun 12;14(6):e0217198. doi: 10.1371/journal.pone.0217198 (PMC6561536; doi:10.1371/journal.pone.0217198)
Supplement: S5 Table — (DOCX) [file pone.0217198.s005.docx]

**S5 Table**: Percentage contribution by the top 70% of the Bray-Curtis dissimilarity matrix for differences between habitat at the three life zones (PPM = ponderosa meadow, PPF = ponderosa forest, MCM = mixed conifer meadow, MCF = mixed conifer forest, SFM = spruce-fir meadow, SFF = spruce-fir forest, PP = Ponderosa, MC = Mixed Conifer, SF = Spruce-fir).

| **PPM - PPF** | | **MCM - MCF** | | **SFM - SFF** | | **PP - MC** | | **MC - SF** | |
| --- | --- | --- | --- | --- | --- | --- | --- | --- | --- |
| **Species** | **Sum** | **Species** | **Sum** | **Species** | **Sum** | **Species** | **Sum** | **Species** | **Sum** |
| Lasioglossum sisymbrii | 9% | Lasioglossum sisymbrii | 16% | Lasioglossum sisymbrii | 21% | Halictus002 | 18% | Ceratina pacifica | 39% |
| Halictus002 | 18% | Ceratina pacifica | 32% | Ceratina arizonensis | 38% | Ceratina pacifica | 30% | Bombus appositus | 56% |
| Perdita002 | 24% | Lasioglossum egregium | 43% | Halyeus | 49% | Perdita003 | 39% | Bombus huntii | 61% |
| Melissodes003 | 30% | Bombus appositus | 49% | Lassioglossum001 | 58% | Protodufourea001 | 44% | Protodufourea001 | 63% |
| Dianthidum001 | 36% | Protodufourea 001 | 55% | Bombus appositus | 66% | Perdita 001 | 51% | Ceratina arizonensis | 67% |
| Diadasia dimanutei | 41% | Melissodes003 | 60% | Bombus huntii | 73% | Lasioglossum egregium | 55% | Bombus ferivdus | 69% |
| Perdita001 | 47% | Bombus huntil | 64% |  |  | Anthidium002 | 61% | Osmia juxta | 71% |
| Perdita003 | 52% | Andrena001 | 68% |  |  | Andrena002 | 68% |  |  |
| Protodufourea001 | 56% | Agapostemon texanus | 71% |  |  |  |  |  |  |
| Andrena002 | 60% |  |  |  |  |  |  |  |  |
| Ceratina pacifica | 64% |  |  |  |  |  |  |  |  |
| Anthophorula001 | 68% |  |  |  |  |  |  |  |  |
| Halyeus001 | 71% |  |  |  |  |  |  |  |  |
