# Supplementary material for: The transition from bee-to-fly dominated communities with increasing elevation and greater forest canopy cover
Source: PLoS One. 2019 Jun 12;14(6):e0217198. doi: 10.1371/journal.pone.0217198 (PMC6561536; doi:10.1371/journal.pone.0217198)
Supplement: S6 Table — (DOCX) [file pone.0217198.s006.docx]

**S6 Table**: Indicator values (IV) and related significance between meadow and forest habitat type at the three life zones (PPM = ponderosa meadow, PPF = ponderosa forest, MCM = mixed conifer meadow, MCF = mixed conifer forest, SFM = spruce-fir meadow, SFF = spruce-fir forest).

| **PPM - PPF** | **IV** | **p.value** |  |
| --- | --- | --- | --- |
| Protodufourea.001 | 0.985 | 0.022 | * |
| **MCM - MCF** | **IV** | **p.value** |  |
| Andrena crinita | 0.995 | 0.003 | ** |
| Perdita.003 | 0.987 | 0.035 | * |
| **SFM - SFF** | **IV** | **p.value** |  |
| Bombus.ferridus | 1 | 0.004 | ** |
| Osmia.juxta | 0.997 | 0.002 | ** |
| Bombus.appositus | 0.994 | 0.003 | ** |
| **PP -MC** | **IV** | **p.value** |  |
| Perdita.001 | 0.481 | 0.019 | * |
| Halictus.002 | 0.473 | 0.017 | * |
| Andrena crinita | 0.447 | 0.031 | * |
| **MC - SF** | **IV** | **p.value** |  |
| Osmia.juxta | 0.597 | 0.004 | ** |
