# Supplementary figures and images for: The transition from bee-to-fly dominated communities with increasing elevation and greater forest canopy cover
Source: PLoS One. 2019 Jun 12;14(6):e0217198. doi: 10.1371/journal.pone.0217198 (PMC6561536; doi:10.1371/journal.pone.0217198)

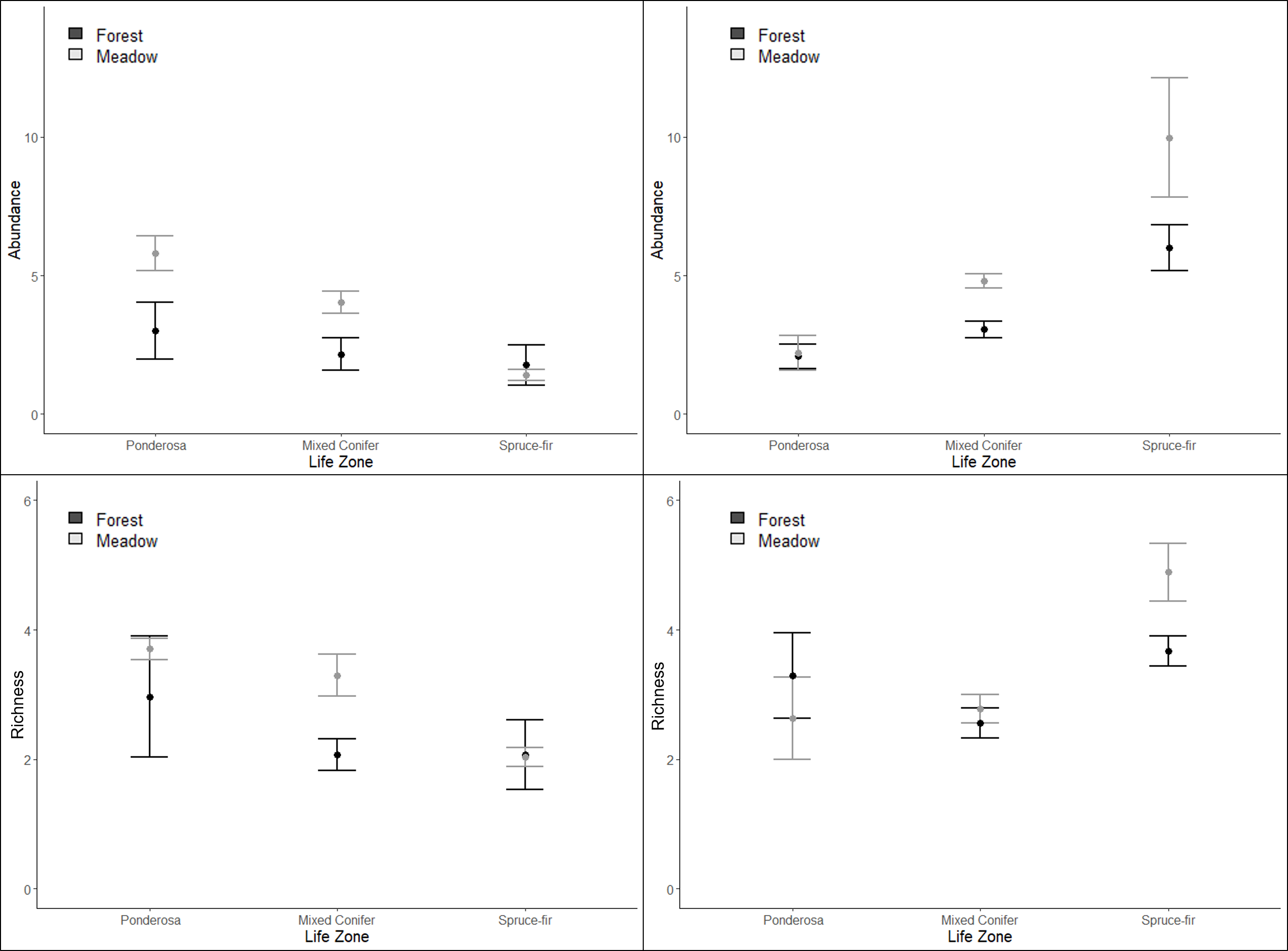

Supplement: S1 Fig — Differences in bee abundance (A) and fly abundance (B) and bee species richness (C) and fly species richness (D) among life zones and between habitats based on average numbers per cup. Bee and fly abundance and species richness among life zones for forest and meadow habitats. (TIFF) [file pone.0217198.s007.tiff]

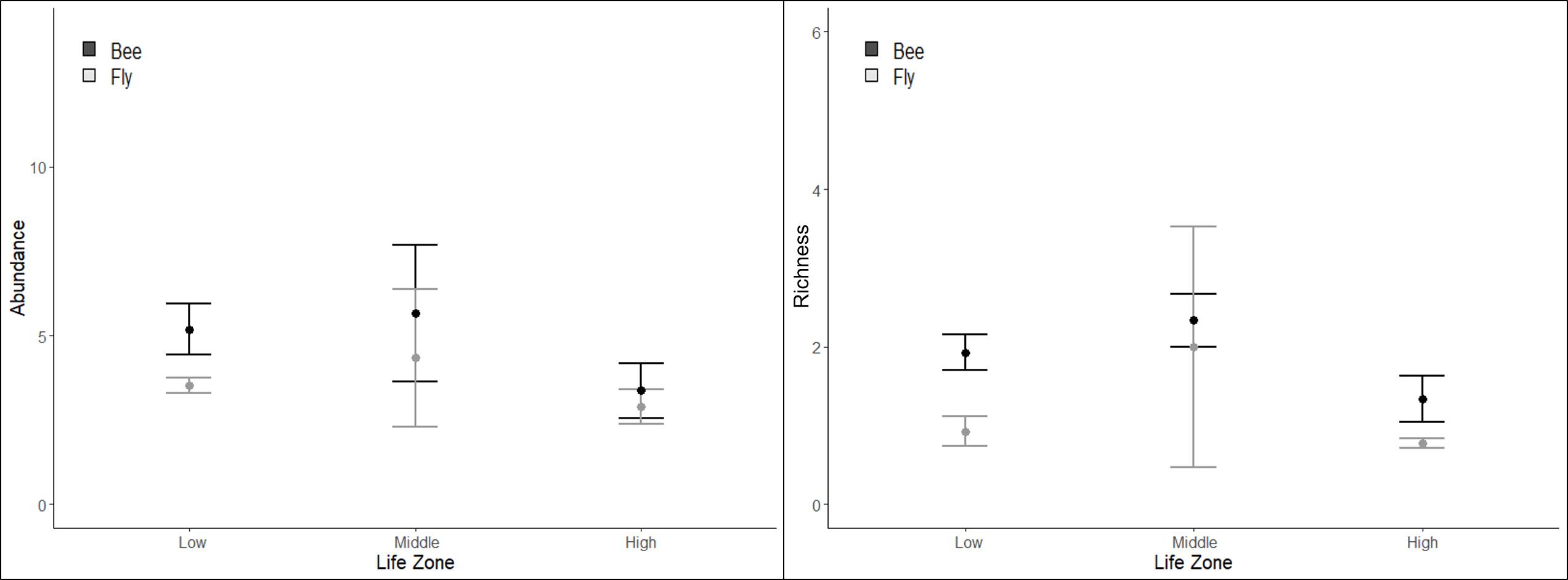

Supplement: S2 Fig — Differences in bee and fly abundance (A) and bee and fly species richness (B) among life zones and between habitats based on average numbers per cup. (TIFF) [file pone.0217198.s008.tiff]
